# Religious practices and changes in health-related quality of life after hospital discharge for an acute coronary syndrome

**DOI:** 10.1186/s12955-019-1218-6

**Published:** 2019-09-03

**Authors:** Hawa O. Abu, David D. McManus, Darleen M. Lessard, Catarina I. Kiefe, Robert J. Goldberg

**Affiliations:** 1 Department of Population and Quantitative Health Sciences, University of Massachusetts Medical School, Worcester, MA 01605 USA; 2 Department of Medicine, Division of Cardiovascular Medicine, University of Massachusetts Medical School, Worcester, MA 01605 USA

**Keywords:** Acute coronary syndrome, Lifestyle, Quality of life, Religion, Spirituality

## Abstract

**Background:**

Religious beliefs and practices influence coping mechanisms and quality of life in patients with various chronic illnesses. However, little is known about the influence of religious practices on changes in health-related quality of life (HRQOL) among hospital survivors of an acute coronary syndrome (ACS). The present study examined the association between several items assessing religiosity and clinically meaningful changes in HRQOL between 1 and 6 months after hospital discharge for an ACS.

**Methods:**

We recruited patients hospitalized for an ACS at six medical centers in Central Massachusetts and Georgia (2011–2013). Participants reported making petition prayers for their health, awareness of intercessory prayers by others, and deriving strength/comfort from religion. Generic HRQOL was assessed with the SF-36®v2 physical and mental component summary scores. Disease-specific HRQOL was evaluated using the Seattle Angina Questionnaire Quality of Life subscale (SAQ-QOL). We separately examined the association between each measure of religiosity and the likelihood of experiencing clinically meaningful increase in disease-specific HRQOL (defined as increases by ≥10.0 points) and Generic HRQOL (defined as increases by ≥3.0 points) between 1- and 6-months post-hospital discharge.

**Results:**

Participants (*n* = 1039) were, on average, 62 years old, 33% were women, and 86% were non-Hispanic White. Two-thirds reported praying for their health, 88% were aware of intercessions by others, and 85% derived strength/comfort from religion. Approximately 42, 40, and 26% of participants experienced clinically meaningful increases in their mental, physical, and disease-specific HRQOL respectively. After adjustment for sociodemographic, psychosocial, and clinical characteristics, petition (aOR:1.49; 95% CI: 1.09–2.04) and intercessory (aOR:1.72; 95% CI: 1.12–2.63) prayers for health were associated with clinically meaningful increases in disease-specific and physical HRQOL respectively.

**Conclusions:**

Most ACS survivors in a contemporary, multiracial cohort acknowledged praying for their health, were aware of intercessory prayers made for their health and derived strength and comfort from religion. Patients who prayed for their health and those aware of intercessions made for their health experienced improvement in their generic physical and disease-specific HRQOL over time. Healthcare providers should recognize that patients may use prayer as a coping strategy for improving their well-being and recovery after a life-threatening illness.

**Electronic supplementary material:**

The online version of this article (10.1186/s12955-019-1218-6) contains supplementary material, which is available to authorized users.

## Background

Approximately 1.1 million adults are hospitalized annually for acute coronary syndrome (ACS) in the United States, constituting a major source of morbidity and mortality [1]. Due to the life-threatening experience of ACS, patients are at increased risk for declining health status and quality of life (QOL) following hospital discharge for ACS [2]. Health-related quality of life (HRQOL), which is characterized by patient’s perception of their well-being, is increasingly being used as an important patient-reported outcome measure for assessing treatment effectiveness and recovery after ACS [3]. Several studies have shown HRQOL to be an independent predictor of subsequent morbidity and mortality in hospital survivors of ACS, with substantial impairment in HRQOL and loss of productive years [4, 5].

Since ACS survivors tend to have impaired HRQOL, management strategies aimed at improving patients’ clinical status and well-being are desirable [6]. While the influence of several sociodemographic and clinical factors on HRQOL among survivors of ACS has been studied [7, 8], very limited research has been conducted to understand the influence of religiosity on HRQOL among hospital survivors of ACS. Religious beliefs and practices provide vital strategies for coping with illness, including strength and optimism in the acute phase of an illness and recovery period [9, 10]. Cross-sectional [11, 12] and longitudinal [13] studies that have examined the relationship between religiosity/spirituality and QOL among patients with various forms of cardiovascular disease (CVD) have, however, demonstrated mixed findings. In our recent systematic review [14], we only identified two earlier studies that examined the influence of patient’s religiosity/spirituality on QOL after ACS. These studies were limited by their small sample size and participants were recruited from a single site with ethnic and religious homogeneity, precluding generalizability to more diverse settings and populations [15, 16].

Using data from a large and socioeconomically diverse cohort of patients hospitalized for ACS, we examined the association between several measures of religiosity and clinically meaningful changes in HRQOL between 1 and 6 months after hospital discharge for ACS.

## Methods

### Study design and population

The Transitions, Risks and Actions in Coronary Events: Center for Outcomes Research and Education (TRACE-CORE) study [17] used a multi-center prospective cohort design to enroll 2174 adults hospitalized for ACS at six medical centers in Central Massachusetts and Georgia (April 2011–May 2013). Two cardiologists independently validated the diagnosis of an ACS based on standard criteria [18]. Each validated case of ACS was categorized as either ST-segment elevation acute myocardial infarction (STEMI), Non-ST segment elevation acute myocardial infarction (NSTEMI), or unstable angina [18]. Trained research personnel abstracted data from electronic medical records on patient’s clinical variables. In addition, we obtained information about patient sociodemographic, psychosocial, and behavioral characteristics from an extensive baseline interview during the index hospitalization in-person or within 72 h of discharge by telephone. Patients participated in additional telephone interviews after hospital discharge. Institutional Review Boards (IRBs) at the participating sites approved the study, and participants provided written informed consent.

### Measures of religiosity

Patients self-reported three measures of religiosity during hospitalization for ACS. The first item asked: “How much is religion a source of strength and comfort to you?”. Response options were “a great deal”, “a little”, “some”, and “none” (referent group). We combined responses of “some” or “little” for analysis. The second item assessed petition prayers for health by asking, “Do you use prayer specifically for your health?” with responses “Yes” or “No” (referent). The third item inquired about intercessory prayers for health: “Do you know of others outside of your family who are praying for your health?” with responses “Yes” or “No” (referent). We evaluated the three measures of religiosity separately in assessing the influence of religious practices on changes in HRQOL.

### Measures of HRQOL

Patient’s generic HRQOL was assessed using the SF-36®v2 Health Survey with norm-based physical and mental well-being component summary scores ranging from 0 to 100; higher scores indicate better generic HRQOL [19]. The physical health dimension assesses bodily pain, physical functioning, role limitations due to physical health, and general well-being [19]. The mental health component assesses emotional role, vitality, social functioning and mental well-being [19]. Clinically meaningful increases in generic HRQOL was defined by changes of ≥3.0 points [20]. Disease-specific HRQOL was assessed with the 3-item quality of life subscale from the Seattle Angina Questionnaire (SAQ-QOL), a validated and reliable measure for patients with coronary heart disease with scores ranging from 0 to 100; higher scores indicate better HRQOL [21]. The SAQ-QOL measures how patients’ physical symptoms (chest pain, chest tightness, or angina) may interfere with their enjoyment of life, and how often they worry about experiencing a heart attack or sudden death [21]. We defined clinically meaningful increases in disease-specific HRQOL by changes of ≥10.0 points [21]. Our rationale for using HRQOL measures obtained at 1-and 6-months post-hospital discharge was, that items in the SF-36®v2 questionnaire reflect patient well-being in the 4-week period after experiencing an ACS, and the 6-month window is relevant in cardiac rehabilitation since most patients typically recover within 12 weeks after an ACS [22]. Inasmuch, we examined if improvement in patient well-being was sustained in the 6-month period after an ACS.

### Participant baseline characteristics

We obtained data on patient’s age, sex, race/ethnicity, level of education, marital status, and employment status. Symptoms of anxiety were measured using the 7-item Generalized Anxiety Disorder questionnaire (GAD-7) [23]. Symptoms of depression were assessed with the 9-item Patient Health Questionnaire (PHQ-9) [24]. The extent to which patients found their lives “uncontrollable, unpredictable and overloading” was assessed with the 4-item Perceived Stress Scale [25]. Social support was measured with 5 items from the Medical Outcomes Social Support Survey Instrument [26]. Patients were asked how confident they were in filling out health forms; they were considered to have low health literacy if they reported having little or no confidence [27]. The 6-item patient Activation Measure (PAM-6) assessed the extent of patient’s knowledge, confidence, and skills in managing their disease [28]. The 11-item Telephone Interview for Cognitive Status (TICS) assessed cognitive status 1-month post-discharge [29].

Lifestyle variables included smoking history and alcohol use. Clinical characteristics included ACS subtype (STEMI, NSTEMI, unstable angina), previously diagnosed co-morbidities, receipt of coronary reperfusion therapy, length of index hospitalization, and referral for cardiac rehabilitation. The Global Registry of Acute Coronary Events (GRACE) risk score (2.0) for long-term mortality was calculated using information on patient’s age, heart rate, systolic blood pressure, elevated cardiac enzymes, ST-segment changes, serum creatinine levels, and the presence of heart failure and cardiac arrest at hospital admission [30].

### Data analysis

We excluded participants with missing information on covariates (race = 10, gender = 19, social support = 22, perceived stress = 35, anxiety symptoms = 27, depression symptoms = 37, GRACE risk score = 39, ACS classification = 46, and referral to cardiac rehabilitation = 41), measures of religiosity (*n* = 41), and patients whose HRQOL measures were not available at 1 (*n* = 163) and 6 (*n* = 650) months post-discharge due to attrition. The analytic sample consisted of 1039 participants of the original study cohort of 2174 patients discharged from participating hospitals after a confirmed ACS.

Descriptive statistics were used to compare patients’ baseline characteristics in relation to the three measures of religiosity. Unpaired t-tests and chi-square compared differences in continuous and categorical variables, respectively. We used paired t-tests to examine differences in mean HRQOL scores from 1 to 6 months after hospital discharge.

Using logistic regression models, we estimated the likelihood of experiencing a clinically meaningful increase in HRQOL between 1 and 6 months after hospital discharge according to the three measures of religiosity. We created separate models to examine the association between each measure of religiosity and changes in generic and disease-specific HRQOL. For multivariable adjustment, potentially confounding variables were included in the regression model based on clinical judgement and factors known to be associated with religiosity and HRQOL. Additional potential confounders were tested to determine if their presence in the model changed the religiosity effect-estimate by more than 10% [31]. We sequentially adjusted for a number of patient associated factors. First, we adjusted for sociodemographic characteristics (age, sex, and race/ethnicity). Subsequently, clinical variables (length of index hospitalization, type of ACS, receipt of coronary reperfusion therapy, referral for cardiac rehabilitation, and GRACE-risk score) were added to the models. Finally, psychosocial measures including symptoms of depression and anxiety and perceived stress, were adjusted for in our models. Since clinical practices and access to healthcare may vary across the participating hospitals, we adjusted for study site in all regression models. Multicollinearity was evaluated and ruled out by using a variance inflation factor (VIF) of ≥3 to detect correlation between covariates. There was no collinearity (VIF = 1.63) between the covariates included in the fully adjusted regression models.

We considered that the relationship between intercessory prayers for health and changes in health-related QOL may differ according to the extent of the patient’s social support system. Inasmuch, we included interaction terms in the initial regression models as product terms between intercessory prayers for health and social support for the respective QOL outcomes. None of the interaction terms in the models were statistically significant. Likelihood ratio tests were used to compare the models with and without interaction terms. We observed no significant differences between the tested models and have provided results from the parsimonious regression models without interaction terms.

In addition to examining the association between religiosity and clinically meaningful changes in disease specific QOL, as assessed by the SAQ-QOL domain, we examined the association between each religiosity item and the other SAQ domains (physical limitation, angina stability, angina frequency, and treatment satisfaction). Each SAQ domain has been independently validated and shown to be reliable and prognostic of cardiovascular outcomes after an ACS [32].

We also conducted sensitivity analysis using inverse probability weighting (IPW) regression models [33] to account for potential selection bias due to differential losses to follow-up among our study participants at 1 and 6 months. The IPW technique assigns a weight to each participant which is equivalent to the inverse probability of remaining in the study at the time points of interest [33]. The censoring weights are applied to the observed population, and censored participants are accounted for, by up-weighting uncensored participants with the same values of observed covariates and measures of exposure [33].

## Results

### Participant baseline characteristics

Study participants (*n* = 1039) were, on average, 62 years old, 33% were women, and 86% were non-Hispanic White. In comparison with included participants, excluded patients were younger, more likely to be of racial/ethnic minority group, had less than high school education and were more likely to be uninsured. Excluded participants reported higher levels of perceived stress, and moderate/severe symptoms of depression and anxiety, had low health literacy, were less likely to have undergone coronary reperfusion therapy during hospitalization, and to have been referred for cardiac rehabilitation (*p* < 0.001). There were no significant differences in the prevalence of the three measures of religiosity between included and excluded study participants.

### Participant characteristics according to measures of religiosity

Study participants reported deriving a great deal (*n* = 524; 50.4%) and little/some (*n* = 362; 34.8%) strength and comfort from religion, while a lesser proportion reported deriving none (*n* = 153; 14.7%). More than one-half of participants prayed for their health (*n* = 616; 59.3%), and a majority were aware of intercessory prayers made for their health (*n* = 918; 88.3%) (Table 1). Participants with affirmative responses to all three measures of religiosity were more likely to be women, non-Hispanic Blacks, report no use of alcohol, were non-smokers, and were less likely to be referred for cardiac rehabilitation than patients who did not respond in the affirmative to the religiosity measures. Severe symptoms of depression and anxiety, and higher levels of perceived stress were more prevalent among those who prayed or were aware of intercessions made for their health.. A greater proportion of participants who prayed for their health were older, more likely to be cognitively impaired, had more previously diagnosed comorbidities, had an NSTEMI, and underwent coronary artery by-pass graft surgery during their index hospitalization than those who did not pray for their health (*p* < 0.05 for all comparisons) (Table 1).

**Table 1:** Baseline sociodemographic, psychosocial and clinical characteristics of hospital survivors of an acute coronary syndrome according to measures of religiosity, TRACE-CORE

Characteristics	Strength and comfort from religion			Petition prayers for health		Intercessory prayers for health	
	A great deal (*n* = 524)	Little/Some (*n* = 362)	None (*n* = 153)	Yes (*n* = 616)	No (*n* = 423)	Yes (*n* = 918)	No (*n* = 121)
Socio-demographic							
Age (mean, years (sd))	63.9 (10.5)	61.1 (10.6)	61.0 (11.3) *	63.2 (10.6)	61.5 (10.9) *	62.3 (10.7)	63.7 (11.0)
Age (years, %)							
< 55	20.8	28.2	31.4	22.7	28.1	25.6	19.8
55–64	29.4	35.6	27.4	30.7	32.2	31.5	29.8
≥ 65	49.8	36.2	41.2	46.6	39.7	42.9	50.4
Women (%) ***	45.4	22.9	17.0	41.9	21.0	35.5	17.4
Married (%)	58.6	68.2	61.4*	61.2	64.1	62.4	62.0
Race/Ethnicity (%) ***							
Non-Hispanic Whites	78.0	92.0	96.0	80.0	93.6	84.6	92.6
Non-Hispanic Blacks	19.7	5.2	2.0	17.4	4.3	13.0	5.0
Hispanics	2.3	2.8	2.0	2.6	2.1	2.4	2.4
Education (≤ high school) (%)	41.8	40.6	38.6	43.3	37.3	41.0	40.5
Unemployed/retired (%)	65.3	53.9	47.1*	63.6	51.3*	58.6	58.7
Uninsured (%)	6.1	6.3	7.8	6.5	6.4	6.4	6.6
Psychosocial (%)							
High perceived stress^†^	44.8	43.1	35.9	44.3	32.2*	47.7	35.9*
Depressive Symptoms^b^							
None	52.5	54.7	60.8	50.8	59.8	52.9	66.1
Mild	29.0	28.4	20.3	28.4	26.2	28.7	19.0
Moderate	12.0	10.5	12.4	13.3	9.0	11.4	12.4
Moderately Severe/Severe	6.4	6.4	6.5	7.5	5.0*	6.0	2.5*
Anxiety Symptoms^c^							
None	55.3	56.6	62.8	53.1	62.4	55.7	66.1
Mild	20.6	22.7	18.3	20.6	21.5	21.7	15.7
Moderate/Severe	24.1	20.7	18.9	26.3	16.1*	22.6	18.2
Low health literacy	38.0	34.4	32.4	37.1	34.3	35.8	38.8
Low social support	4.2	2.8	7.2	4.2	4.0	3.5	9.1*
Cognitive impairment ^d^ ***	18.9	10.8	5.2	19.2	6.6	15.0	6.6
Patient Activation Level (%)							
1: Disengaged (lowest)	19.3	22.4	19.0	20.4	20.1	19.9	23.2
2: Aware	33.6	34.2	47.7	35.1	37.1	35.6	38.0
3: Taking Action	22.9	18.5	22.2	20.1	22.9	21.5	19.8
4: Maintaining Behaviors	24.2	24.9	11.1*	24.4	19.9	23.0	19.0
Behavioral and Clinical							
Length of stay, ≥3 days (%) ***	42.9	66.0	77.8	52.6	31.2	45.5	31.4
Alcohol use (%) ***							
No alcohol use	49.8	32.3	32.7	47.2	32.4	42.5	31.4
Rare/occasional	32.8	40.6	39.2	33.1	41.4	36.8	33.9
Moderate/heavy	17.4	27.1	28.1	19.7	26.2	20.7	34.7
Smoking status (%) ***							
Non-smoker	35.7	26.5	29.4	34.9	26.7	32.7	23.1
Prior smoker	49.4	50.8	46.4	49.2	49.9	49.7	47.9
Current smoker	14.9	22.7	24.2	15.9	23.4	17.6	29.0
GRACE risk score, mean (SD)^e^	99.6 (26.4)	93.1 (26.3)	92.3 (26.9) *	98.2 (26.1)	93.5 (27.1) *	96.2 (26.9)	96.9 (25.6)
Co-morbidities at admission (%)							
Chronic kidney disease	11.4	8.6	7.2	11.8	9.9	13.8	8.3
Congestive heart failure	13.7	9.7	8.5	13.8	8.3*	11.8	9.9
Diabetes mellitus	33.8	28.7	28.8	32.0	30.3	31.5	29.7
Hypertension	80.0	69.6	69.3*	77.9	70.2*	75.4	70.2
Stroke	5.7	4.7	0.6*	6.2	2.4*	4.9	2.5
Type of ACS (%)							
Unstable Angina	33.4	28.2	26.8	33.3	19.4	30.6	30.6
NSTEMI	54.6	55.5	53.4	55.4	53.9	54.8	54.5
STEMI	12.0	16.3	19.6	11.3	26.7*	14.6	14.9
Reperfusion therapy (%)							
Medical treatment	20.8	16.0	18.3	20.1	16.8	19.2	15.7
PCI	64.5	71.8	71.9	65.1	72.6	67.0	76.9
CABG	14.7	12.2	9.8	14.8	10.6*	13.8	7.4
Cardiac rehabilitation referral (%) ***	33.8	49.4	55.6	35.9	52.0	40.7	55.3

Abbreviations: *NSTEMI* Non-ST segment elevation myocardial infarction, *STEMI* ST segment elevation myocardial infarction, *PCI* percutaneous coronary intervention, *CABG* coronary artery by-pass graft

* *P* < 0.05 across response categories for respective religiosity measure; ^a^ Cohen’s Perceived Stress Scale Score (≥4 median, high perceived stress); *** *P* < 0.05 across response categories for all 3 religiosity measures; ^b^ PHQ-9 Patient Health Questionnaire 9 item score (5–9 mild; 10–14 moderate; 15–19 moderately severe; and ≥ 20 severe depression)

^c^ GAD-7 General Anxiety Disorder 7 item score (5–9 mild; 10–14 moderate; ≥15 severe anxiety); ^d^TICS Telephone Interview for Cognitive Status Score (≤ 28 impaired)

^e^ GRACE risk score estimates mortality risk at 1 and 3 years after ACS admission. Score ranges from 0 to 263, higher scores worse. Derived from data on age, systolic blood pressure, ST segment changes, cardiac biomarkers, serum creatinine or history of renal dysfunction, Killip class or diuretic use, cardiac arrest during hospitalization for ACS

### Generic mental and physical HRQOL

Overall, study participants experienced a significant increase in mean MCS scores from 50.7 at 1 month to 52.5 at 6 months post-hospital discharge (*p* < 0.001); 42.3% of patients experienced a clinically meaningful increase in their MCS score (i.e. ≥3.0 points). A significantly higher proportion of participants who reported deriving a great deal or little/some strength and comfort from religion experienced clinically meaningful increases in their MCS-HRQOL score compared with those who derived none (47.0% vs 38.6% vs 34.6%). Similarly, a higher proportion of patients who were aware of intercessions made for their health experienced a clinically meaningful increase in their MCS-HRQOL score (Table 2).

**Table 2:** Generic and disease specific HRQOL scores, mean change and clinically meaningful increase in survivors of acute coronary syndrome after 1 to 6 months for hospital discharge

	Strength and comfort from religion			Petition prayers for health		Intercessory prayers for health	
	A great deal (*n* = 524)	Little/Some (*n* = 362)	None (*n* = 153)	Yes (*n* = 616)	No (*n* = 423)	Yes (*n* = 918)	No (*n* = 121)
Generic SF36v2 MCS							
1 month post-discharge, Mean (SD)	50.1 (11.4)	50.9 (10.8)	52.1 (11.5)	49.9 (11.3)	51.9 (10.9)*	50.5 (11.2)	51.8 (11.2)
6 months post-discharge, Mean (SD)	52.2 (11.4)	52.5 (10.5)	53.3 (10.6)	51.8 (11.4)	53.4 (10.4)*	52.3 (11.2)	53.5 (9.2)
Change in MCS-QOL score, Mean (SD)	2.1 (10.1)	1.6 (9.0)	1.1 (9.1)	1.9 (9.7)	1.6 (9.3)	1.8 (9.7)	1.7 (8.4)
Clinically meaningful increase (%)	47.0	38.6	34.6*	44.5	39.1	43.4	33.9*
Generic SF36v2 PCS							
1 month post-discharge, Mean (SD)	41.0 (10.4)	43.1 (9.9)	44.6 (10.3)*	41.1 (10.5)	43.9 (9.7)*	41.9 (10.3)	45.3 (9.7)*
6 months post-discharge, Mean (SD)	42.4 (11.6)	45.0 (11.5)	46.4 (10.8)*	42.9 (11.6)	45.3 (11.4)*	43.6 (11.5)	45.7 (11.6)*
Change in PCS-QOL score, Mean (SD)	1.4 (8.6)	1.9 (7.9)	1.8 (9.2)	1.8 (8.3)	1.4 (8.6)	1.8 (8.5)	0.5 (8.1)
Clinically meaningful increase (%)	38.0	42.9	40.1	39.9	40.2	41.2	30.8*
Disease Specific SAQ-QOL							
1 month post-discharge, Mean (SD)	74.2 (23.7)	76.4 (22.6)	77.4 (21.8)	72.6 (23.7)	79.5 (21.5)*	74.8 (23.2)	80.2 (21.8)
6 months post-discharge, Mean (SD)	79.1 (21.7)	81.0 (21.7)	83.2 (17.9)	78.6 (22.2)	82.9 (19.3)*	79.9 (21.4)	83.5 (19.5)
Change in SAQ-QOL score, Mean (SD)	4.8 (20.6)	4.6 (18.8)	5.7 (20.0)	5.9 (20.2)	3.3 (19.3)	5.1 (19.9)	3.4 (19.5)
Clinically meaningful increase (%)	28.2	23.2	28.1	29.9	21.5*	27.3	19.8

* *P* < 0.05 across response categories for respective religiosity measure

Note: The SF36v2 MCS (mental component summary) and SF36v2PCS (physical component summary) scores are norm-based ranging from 0 to 100 with a mean of 50 (SD = 10) in the US general population and higher scores indicate better generic health related quality of life (HRQOL). A clinically meaningful increase was defined as ≥3.0 points change in generic HRQOL from 1 to 6 months post discharge for an acute coronary syndrome

The Seattle Angina Questionnaire Quality of Life (SAQ-QOL) subscale contains 3 items scored on a scale of 0–100 with higher scores indicative of better disease-specific health related quality of life (HRQOL). A clinically meaningful increase was defined as ≥10.0 points change in disease specific HRQOL from 1 to 6 months post discharge for an acute coronary syndrome

For all patients, the average PCS scores increased by 1.6 points from 42.3 at 1 month to 43.9 at 6 months post-hospital discharge (*p* < 0.001); less than half (40%) experienced a clinically meaningful increase in their PCS score (i.e. ≥3.0 points). A significantly higher proportion of patients who had intercessions made for their health experienced a clinically meaningful increase in their PCS-HRQOL score compared with those unaware of intercessions made for their health (Table 2).

### Disease-specific HRQOL

Overall, the mean disease-specific HRQOL scores increased from 75.5 at 1 month to 80.3 at 6 months post-discharge (*p* < 0.001); 26.5% of patients experienced a clinically meaningful increase in their disease-specific HRQOL score (i.e. ≥10.0 points).. A significantly higher proportion of patients who prayed for their health experienced a clinically meaningful increase in their disease-specific HRQOL score than those who did not pray for their health (Table 2).

### Association between religiosity and changes in generic and disease-specific HRQOL

After adjusting for several sociodemographic, clinical, and psychosocial characteristics, patients aware of intercessory prayers made for their health experienced a clinically meaningful increase in their generic physical HRQOL between 1- and 6-months post-hospital discharge compared with those unaware of intercessions made for their health (adjusted Odd Ratio [aOR]:1.72; 95% CI: 1.12–2.63). Similarly, patients who prayed for their health were more likely to experience a clinically meaningful increase in their disease-specific HRQOL (Table 3).

**Table 3:** Association between religiosity measures and clinically meaningful increase in generic and disease specific HRQOL among survivors of acute coronary syndrome after 1 to 6 months for hospital discharge

Religiosity Measures	Clinically meaningful increase in MCS-QOL		Clinically meaningful increase in PCS-QOL		Clinically meaningful increase in SAQ-QOL	
	Unadjusted model OR (95% CI)	Fully adjusted model^a^ OR (95% CI)	Unadjusted model OR (95% CI)	Fully adjusted model^a^ OR (95% CI)	Unadjusted model OR (95% CI)	Fully adjusted model^a^ OR (95% CI)
Strength and comfort from religion						
A great deal	1.67 (1.15–2.44)	1.47 (0.97–2.21)	0.92 (0.63–1.32)	1.06 (0.70–1.59)	1.01 (0.67–1.50)	0.95 (0.61–1.48)
Little/Some	1.19 (0.80–1.76)	1.14 (0.76–1.72)	1.12 (0.76–1.65)	1.17 (0.78–1.74)	0.77 (0.50–1.19)	0.74 (0.48–1.15)
None	Ref	Ref	Ref	Ref	Ref	Ref
Petition Prayers for health						
Yes	1.25 (0.97–1.60)	1.06 (0.80–1.39)	0.99 (0.76–1.27)	1.16 (0.88–1.54)	1.55 (1.16–2.07)	**1.49 (1.09–2.04)**
No	Ref	Ref	Ref	Ref	Ref	Ref
Intercessory Prayers for health						
Yes	1.50 (1.00–2.23)	1.31 (0.87–1.99)	1.57 (1.04–2.37)	**1.72 (1.12–2.63)**	1.52 (0.95–2.43)	1.44 (0.88–2.33)
No	Ref	Ref	Ref	Ref	Ref	Ref

^a^Adjusted for sex, race/ethnicity, perceived stress, symptoms of depression and anxiety, length of index hospitalization, type of ACS, GRACE-risk score, receipt of reperfusion therapy, referral for cardiac rehabilitation, and study sites

**Bold text**: Statistically significant results from the fully adjusted regression models

In examining the association between religiosity and the other SAQ domains, participants who reported petition or intercessory prayers for their health had clinically meaningful improvement in their physical limitation compared with those without petition or intercessory prayers made for their health, respectively. Participants who reported receiving strength and comfort from religion were less likely to experience clinical improvement in their angina symptoms. Lastly, those who reported petition prayers for their health had greater clinical improvement in the frequency of their angina symptoms (Additional file 1).

The results from our sensitivity analysis using IPW regression models generated effect estimates consistent with those obtained from the unweighted regression models (Additional file 2), reducing the likelihood of potential selection bias.

## Discussion

In this prospective study we examined the influence of religious practices on clinically meaningful changes in generic physical and mental, and disease-specific HRQOL between 1- and 6-months after hospital discharge for ACS. Most participants acknowledged praying for their health, were aware of intercessory prayers made for their health, and derived strength and comfort from religion. However, less than one-half of patients experienced clinically meaningful increases in their generic mental and physical HRQOL, and only one in four patients had an increase in their disease-specific HRQOL between 1- and 6-months post-discharge. After accounting for several potentially confounding sociodemographic, clinical, and psychosocial variables, participants aware of intercessions made for their health and those who prayed for their health were more likely to experience clinically meaningful increases in their generic physical and disease-specific HRQOL over time.

### Extent of religious practices for health

The high levels of religious engagement for health reported by our study participants is consistent with the findings in other patient populations. In a sample of 151 patients who underwent CABG surgery, 68% reported praying for their health to cope in the post-operative period [34]. In a nationally representative sample of 2262 American adults with a history of cancer, 69% reported using prayers for their own health [35]. In a qualitative study of 9 patients with an acute myocardial infarction in Iran, participants mentioned that trust in God, praying for their health, and deriving strength from their religion provided a coping mechanism in dealing with their life-threatening illness and aided their recovery process [36]. Our results and those from other studies suggest that in stressful life circumstances such as an acute illness, patients may utilize their beliefs including praying for their health or seeking strength from God to provide meaning, hope, and support in dealing with their illness [37]. To ensure a holistic approach in patient management, healthcare providers need to acknowledge the role of patient’s religious beliefs in influencing their recovery from illness, perception of their well-being, and engagement with their healthcare.

### Religiosity and changes in generic physical and mental HRQOL

Overall, our study participants had lower physical HRQOL scores in comparison with their mental HRQOL scores at 1- and 6-months post-discharge and were more likely to experience greater improvement in their mental than in their physical well-being. Our findings align with previous studies reporting greater impairment in physical functioning compared with mental functioning after hospital discharge for an ACS [38, 39]. The distressing physical symptoms associated with ACS such as fatigue, dyspnea, or chest pain have been shown to negatively impact patient’s physical well-being to a greater extent than their mental well-being [38]. When faced with such a potentially life-threatening illness, in addition to seeking medical intervention, patients may pray for their healing and request intercessory prayers from others for their health. The awareness of being prayed for could foster a feeling of spiritual support, improved well-being, greater resilience, and the ability to cope with their physical symptoms [40, 41]. In support of this claim, we found that study participants aware of intercessions made for their health were more likely to experience clinically meaningful increases in their physical HRQOL than those unaware of intercessions made for their health, even after adjustment for various potential confounders including receipt of coronary reperfusion therapy and referral to cardiac rehabilitation.

In the present study, patients who endorsed all three measures of religiosity were more likely to experience clinically meaningful increases in their mental HRQOL. However, this association was rendered non-statistically significant in the fully adjusted regression models. Prior studies have shown a positive influence of religiosity on mental health with greater life satisfaction, and reduced feelings of hopelessness when faced with challenging circumstances [42, 43]. However, the influence of religiosity on mental wellbeing may be cumulative over the life course as one develops faith in God and confidence in their ability to deal with challenging situations [44]. A possible reason why our measures of religiosity were not significantly associated with changes in mental HRQOL may be due to our inability to account for time varying confounding since we assessed religiosity at only one point in time, and not serially. Our measures did not adequately capture patient’s religiosity before their illness and whether they increased or decreased their religious practices in the months after hospital discharge. This underscores the need for future longitudinal studies to better understand how changes in religious practices may influence long-term health outcomes using a life-course approach.

### Religiosity and changes in disease-specific HRQOL

Consistent with our hypothesis, we found that patients who prayed for their health experienced clinically meaningful increases in their disease-specific HRQOL. Praying for one’s health during recovery from illness has been associated with high levels of optimism, making meaning of an illness experience, and fosters well-being [45]. Our results are consistent with reports from prior studies [46, 47]. Among cardiac patients undergoing bypass surgery, positive aspects of religious coping such as seeking spiritual support and healing from God, was associated with less distress and fatigue [46]. Similarly, in those living with HIV/AIDS, praying at least once daily was associated with improved quality of life and emotional well-being [47]. It is important to note, however, that with religious struggle and negative religious coping where those who pray for their health focus solely on being healed, this may lead to increased levels of anxiety and negatively impact their recovery and wellbeing [48]. Healthcare providers need to acknowledge the possibility of negative religious coping and refer to hospital chaplains to assist patients in overcoming such religious struggle or negative coping detrimental to their health.

### Study strengths and limitations

The present study has several strengths. To the best of our knowledge, this is the first contemporary inquiry into the influence of prayers for health and strength/comfort from religion on changes in HRQOL among hospital survivors of ACS from a large cohort with racial and sociodemographic diversity. Our measures of religiosity directly captured patient’s utilization of their religious beliefs and practices during recovery from a potentially life-threatening cardiovascular disease. In addition, we adjusted for several psychological factors known to impact quality of life including symptoms of depression, anxiety, and perceived stress [49]. Despite adjusting for several important potential confounders, there remains the likelihood of residual and unmeasured confounding given our observational study design. In addition, our findings of high religious engagement among our study participants in the immediate period of a life-threatening illness may not be generalizable to more chronically ill patients who may have adapted to their illness. Despite these limitations, our study demonstrates that praying for one’s recovery might influence their well-being, hence buttressing the need for a holistic approach in patient management, addressing the needs of the body, mind, and spirit for a healthier being.

## Conclusions

Most ACS survivors in a contemporary, multiracial cohort acknowledged praying for their health, were aware of others praying for their health, and reported deriving strength and comfort from religion. Petition and intercessory prayers for health were associated with greater improvement in disease-specific and generic physical HRQOL over a 6-month follow-up period. Our findings are consistent with a recent scientific statement by the American Heart Association (AHA) which suggest that meditation practices, including prayers, are beneficial in improving cardiovascular health outcomes with notably low costs of implementation [50]. This AHA expert panel also recommended that meditation practices be considered as an adjunct to guideline directed cardiovascular risk reduction, especially for patients interested in lifestyle modification.

## Additional files

Additional file 1: Association between religiosity measures and clinically meaningful increase in the other SAQ domains among survivors of acute coronary syndrome after 1 to 6 months for hospital discharge. (DOCX 13 kb)

Additional file 2: Inverse probability weighted regression estimates on the association between religiosity measures and clinically meaningful increase in generic and disease specific HRQOL among survivors of acute coronary syndrome after 1 to 6 months for hospital discharge. (DOCX 13 kb)

## Data Availability

The datasets used and analyzed during the current study are available from the corresponding author on reasonable request.

## Abbreviations

ACS: Acute Coronary Syndrome(s)
AHA: American Heart Association
CABG: Coronary Artery Bypass Graft
CVD: Cardiovascular disease
GRACE: Global Registry of Acute Coronary Events
HRQOL: Health-Related Quality of Life
IPW: Inverse Probability Weights
NSTEMI: Non-ST Elevated Myocardial Infarction
PAM-6: 6-item Patient Activation Measure
PCI: Percutaneous Coronary Intervention
PHQ-9: Patient Health Questionnaire – 9
QOL: Quality of Life
SF-36: 36-Item Short Form Survey (SF-36)
STEMI: ST-Elevated Myocardial Infarction
TICS: Telephone Interview for Cognitive Status
TRACE-CORE: Transitions, Risks, and Actions in Coronary Events – Center for Outcomes Research and Education
