# Supplementary material for: Religious practices and changes in health-related quality of life after hospital discharge for an acute coronary syndrome
Source: Health Qual Life Outcomes. 2019 Sep 3;17:149. doi: 10.1186/s12955-019-1218-6 (PMC6724337; doi:10.1186/s12955-019-1218-6)
Supplement: Supplementary file 1 — Association between religiosity measures and clinically meaningful increase in the other SAQ domains among survivors of acute coronary syndrome after 1 to 6 months for hospital discharge. (DOCX 13 kb) [file 12955_2019_1218_MOESM1_ESM.docx]

Additional file 1. Association between religiosity measures and clinically meaningful increase in the other SAQ domains among survivors of acute coronary syndrome after 1 to 6 months for hospital discharge

| Religiosity Measures | Clinically meaningful increase in  SAQ-Physical limitation | | Clinically meaningful increase in  SAQ-Angina Stability | | Clinically meaningful increase in  SAQ-Angina Frequency | | Clinically meaningful increase in  SAQ-Treatment Satisfaction | |
| --- | --- | --- | --- | --- | --- | --- | --- | --- |
|  | Unadjusted model  OR (95% CI) | Fully adjusted model*  OR (95% CI) | Unadjusted model  OR (95% CI) | Fully adjusted model*  OR (95% CI) | Unadjusted model  OR (95% CI) | Fully adjusted model*  OR (95% CI) | Unadjusted model  OR (95% CI) | Fully adjusted model*  OR (95% CI) |
| Strength and comfort from religion |  |  |  |  |  |  |  |  |
| A great deal | 1.81 (1.09-2.74) | 1.62 (0.94-2.78) | 0.71 (0.41-1.21) | 0.50 (0.26-0.92) | 1.42 (0.94-2.14) | 1.20 (0.76-1.89) | 0.96 (0.60-1.53) | 0.87 (0.52-1.46) |
| Little/Some | 1.63 (0.97-2.73) | 1.59 (0.93-2.71) | 0.37 (0.19-0.70) | 0.31 (0.16-0.61) | 0.89 (0.57-1.38) | 0.84 (0.53-1.32) | 0.79 (0.47-1.30) | 0.77 (0.46-1.29) |
| None | Ref | Ref | Ref | Ref | Ref | Ref | Ref | Ref |
| Petition Prayers for health |  |  |  |  |  |  |  |  |
| Yes | 1.65 (1.19-2.28) | 1.50 (1.06-2.12) | 1.23 (0.79-1.91) | 0.99 (0.61-1.60) | 1.67 (1.25-2.22) | 1.41 (1.04-1.93) | 1.22 (0.87-1.71) | 1.14 (0.79-1.65) |
| No | Ref | Ref | Ref | Ref | Ref | Ref | Ref | Ref |
| Intercessory Prayers for health |  |  |  |  |  |  |  |  |
| Yes | 2.03 (1.15-3.62) | 1.93 (1.06-3.50) | 1.72 (0.77-3.81) | 1.47 (0.65-3.32) | 1.62 (1.01-2.60) | 1.39 (0.86-2.27) | 0.90 (0.54-1.47) | 0.83 (0.50-1.39) |
| No | Ref | Ref | Ref | Ref | Ref | Ref | Ref | Ref |
| *Adjusted for sex, race/ethnicity, perceived stress, symptoms of depression and anxiety, social support, length of index hospitalization, type of ACS, GRACE-risk score, receipt of reperfusion therapy, referral for cardiac rehabilitation, and study site. | | | | | | | | |
