# Supplementary material for: Religious practices and changes in health-related quality of life after hospital discharge for an acute coronary syndrome
Source: Health Qual Life Outcomes. 2019 Sep 3;17:149. doi: 10.1186/s12955-019-1218-6 (PMC6724337; doi:10.1186/s12955-019-1218-6)
Supplement: Supplementary file 2 — Inverse probability weighted regression estimates on the association between religiosity measures and clinically meaningful increase in generic and disease specific HRQOL among survivors of acute coronary syndrome after 1 to 6 months for hospital discharge. (DOCX 13 kb) [file 12955_2019_1218_MOESM2_ESM.docx]

Additional file 2. Inverse probability weighted regression estimates on the association between religiosity measures and clinically meaningful increase in generic and disease specific HRQOL among survivors of acute coronary syndrome after 1 to 6 months for hospital discharge.

| Religiosity Measures | Clinically meaningful increase in  MCS-QOL | | Clinically meaningful increase in  PCS-QOL | | Clinically meaningful increase in SAQ-QOL | |
| --- | --- | --- | --- | --- | --- | --- |
|  | Unadjusted model  OR (95% CI) | Fully adjusted model*  OR (95% CI) | Unadjusted  model  OR (95% CI) | Fully adjusted model*  OR (95% CI) | Unadjusted model  OR (95% CI) | Fully adjusted model*  OR (95% CI) |
| Strength and comfort from religion |  |  |  |  |  |  |
| A great deal | 1.62 (1.09-2.41) | 1.48 (0.95-2.31) | 0.79 (0.53-1.16) | 0.91 (0.59-1.40) | 1.05 (0.69-1.59) | 1.01 (0.67-1.66) |
| Little/Some | 1.06 (0.70-1.61) | 1.03 (0.67-1.59) | 1.05 (0.70-1.58) | 1.12 (0.74-1.71) | 0.77 (0.49-1.59) | 0.74 (0.46-1.17) |
| None | Ref | Ref | Ref | Ref | Ref | Ref |
| Petition Prayers for health |  |  |  |  |  |  |
| Yes | 1.30 (0.99-1.70) | 1.12 (0.84-1.51) | 0.89 (0.68-1.16) | 1.07 (0.80-1.43) | 1.54 (1.13-2.09) | 1.50 (1.09-2.06) |
| No | Ref | Ref | Ref | Ref | Ref | Ref |
| Intercessory Prayers for health |  |  |  |  |  |  |
| Yes | 1.53 (1.01-2.32) | 1.35 (0.88-2.06) | 1.53 (1.00-2.33) | 1.70 (1.10-2.61) | 1.41 (0.87-2.29) | 1.35 (0.82-2.23) |
| No | Ref | Ref | Ref | Ref | Ref | Ref |
| *Adjusted for sex, race/ethnicity, perceived stress, symptoms of depression and anxiety, length of index hospitalization, type of ACS, GRACE-risk score, receipt of reperfusion therapy and study sites. | | | | | | |
